# Cognitive Aging and the Hippocampus in Older Adults

**DOI:** 10.3389/fnagi.2016.00298

**Published:** 2016-12-08

**Authors:** Andrew O’Shea, Ronald A. Cohen, Eric C. Porges, Nicole R. Nissim, Adam J. Woods

**Affiliations:** ^1^Center for Cognitive Aging and Memory, McKnight Brain Institute, Department of Clinical and Health Psychology, University of FloridaGainesville, FL, USA; ^2^Department of Neuroscience, University of FloridaGainesville, FL, USA

**Keywords:** cognitive aging, hippocampus, MoCA, NIH toolbox, structural magnetic resonance imaging

## Abstract

The hippocampus is one of the most well studied structures in the human brain. While age-related decline in hippocampal volume is well documented, most of our knowledge about hippocampal structure-function relationships was discovered in the context of neurological and neurodegenerative diseases. The relationship between cognitive aging and hippocampal structure in the absence of disease remains relatively understudied. Furthermore, the few studies that have investigated the role of the hippocampus in cognitive aging have produced contradictory results. To address these issues, we assessed 93 older adults from the general community (mean age = 71.9 ± 9.3 years) on the Montreal Cognitive Assessment (MoCA), a brief cognitive screening measure for dementia, and the NIH Toolbox-Cognitive Battery (NIHTB-CB), a computerized neurocognitive battery. High-resolution structural magnetic resonance imaging (MRI) was used to estimate hippocampal volume. Lower MoCA Total (*p* = 0.01) and NIHTB-CB Fluid Cognition (*p* < 0.001) scores were associated with decreased hippocampal volume, even while controlling for sex and years of education. Decreased hippocampal volume was significantly associated with decline in multiple NIHTB-CB subdomains, including episodic memory, working memory, processing speed and executive function. This study provides important insight into the multifaceted role of the hippocampus in cognitive aging.

## Introduction

From the discovery of its role in episodic memory following bilateral resection in patient “HM” to the discovery of its role in symptoms of Alzheimer’s disease (AD), the hippocampus is considered a structure fundamental for human cognition (Scoville and Milner, 1957; Squire, 1992; Jack et al., 1999). In addition to its well-documented role in memory function, recent research demonstrates that the hippocampus also plays a role in executive function, processing speed, intelligence, path integration and spatial processing (Reuben et al., 2011; Papp et al., 2014; Yamamoto et al., 2014). Each of these cognitive processes is shown to decline in the context of cognitive aging, in the absence of neurodegenerative diseases or neurological injury (Salthouse, 2010). Thus, understanding how change in hippocampal structure impacts cognition in the context of aging may prove important for identifying: (a) critical neural underpinnings of the cognitive aging process; and (b) intervention targets for combating cognitive aging.

Most of our knowledge of hippocampal structure-function relationships in humans is based on the findings in various disease states or following resection of the medial temporal lobes resulting in gross memory disturbance. Models of structure-function relationships in non-human animals have highlighted the hippocampus as a spatial map, crucial for navigation and spatial memory (O’Keefe and Dostrovsky, 1971; O’Keefe, 1979). In addition, recent functional magnetic resonance imaging (MRI) findings have provided insight into the functional role of the hippocampus in various cognitive abilities beyond episodic and spatial memory (Eldridge et al., 2000; Iaria et al., 2007; Woods et al., 2013). However, the impact of subtle changes in hippocampal structure in the context of normal aging, in the absence of neurodegenerative or other disease states, remains poorly understood.

Individuals with diagnoses of mild cognitive impairment (MCI) and dementia have smaller hippocampi than age-matched controls in numerous MRI studies (Shi et al., 2009). Hippocampal atrophy is considered a hallmark of Alzheimer’s disease (AD) (Jack et al., 1999). Premorbid hippocampal volume in patients with MCI predicts future conversion to AD (Jack et al., 1999). Thus, change in the structure of the hippocampus appears to play an important role in dementia. However, hippocampal volume is also well-documented to decline in normal aging (Raz et al., 2005). Yet, the functional consequences of this age-related volumetric loss is not well characterized in the context of aging in the absence of neurodegenerative disease. While changes in cognitive scores on dementia screening and cognitive assessment measures in patients with MCI and AD are associated with smaller hippocampal volume, it is unclear whether these findings are unique to dementia/disease states or extend to more subtle variations in hippocampal structure from normal aging.

The few studies that have investigated cognitive aging and the hippocampus have produced results that contrast significantly with prior research in neurological and neurodegenerative disease (Van Petten, 2004; Paul et al., 2011; Colom et al., 2013). For example, Van Petten (2004), in a meta-analysis, reported that the relationship between hippocampal size and episodic memory were weak. These inconsistencies between aging and disease-related findings highlight the need for further investigation of the role of the hippocampus in cognitive aging. Understanding the relationship between hippocampal structure and function in cognitive aging may have predictive value for identifying persons at higher risk for future cognitive decline, cognitive frailty and conversion to MCI (Woods et al., 2013). The prevalence of older adults is expected to accelerate over the coming decades. With this shift in the age of the world population comes an increase in the number of people that will suffer from MCI and other neurodegenerative disorders. Thus, there is a pressing need to identify predictive markers of decline. However, pursuit of such markers is difficult, if not impossible, without first understanding the normal variation present in the aging brain, as well as the overall structure-function relationship between the hippocampus and different components of cognitive function.

In the current study, we sought to examine the relationship between hippocampal volume and a commonly administered dementia-screening tool and a comprehensive cognitive battery in a cohort of 93 older adults without neurological injury, neurodegenerative disease or major psychiatric illness to: (1) better understand the structure-function relationship between the hippocampus and cognitive aging; and (2) to providing a foundation for development of predictive biomarkers by characterizing the sensitivity of commonly administered MCI screening and cognitive assessment tools to age-related structural changes in the hippocampus. We specifically examined the relationship between the Montreal Cognitive Assessment (MoCA) and the NIH Toolbox Cognitive Battery (NIHTB-CB). The MoCA is a brief (10 min) screening tool for MCI (Nasreddine et al., 2005), whereas the NIH toolbox cognitive assessment is a brief comprehensive computerized cognitive battery (~60 min) consisting of tests to assess executive function, attention, episodic memory, language, processing speed and working memory. These measures are sub-divided into two composite cognitive scores comprising cognitive abilities that change with age (fluid cognitive function) or remain stable over time (crystalized cognitive functions). The delineation of a two-factor model (a fluid factor and a crystalized factor) instead of a single general intelligence factor is valuable when studying cognitive aging due to differences in the age curves of fluid and crystalized abilities (Cattell, 1987). Mungas et al. (2014) found a two-factor solution fit the NIHTB-CB validation data better than a single general intelligence factor; however, the best fitting model was a five-factor solution comprised of the following: reading, vocabulary, episodic memory, working memory and executive function/processing speed. An extension of the two-factor, fluid and crystalized model, the Cattell-Horn-Carroll (CHC) theory of cognition extends the factors of general intelligence to nine broad abilities (fluid reasoning, comprehension-knowledge, short-term memory, visual processing, auditory processing, long-term storage and retrieval, cognitive processing speed, quantitative knowledge and reading and writing; McGrew, 2009). The CHC taxonomy may provide a more thorough description of individual domains of the NIHTB-CB. However, a single NIHTB-CB task would likely incorporate multiple factors of the CHC model, rather than representing distinct entities.

We hypothesized that older adults with smaller hippocampal volumes would evidence lower performance on both the MoCA and NIHTB fluid cognition scores. In contrast, language-based cognitive abilities (i.e., crystalized cognition) would not change as a function of hippocampal volume. Furthermore, we hypothesized that hippocampal volume would be most strongly associated with performance on the memory domain of the MoCA and NIHTB. In addition, we also predicted that smaller hippocampal volume would be associated with slower processing speed and poorer executive functions. These data would not only support the role of the hippocampus in cognition as shown in prior research on neurodegenerative disease and neurological disease states, but also extend these findings to cognitive aging. Furthermore, these data would provide a strong foundation for development of predictive hippocampal biomarkers for future decline in longitudinal cohorts by characterizing cognitive aging in the hippocampus in the absence of neurological and neurodegenerative disease.

## Materials and Methods

### Participants

Ninety-three older adults (60% female) were recruited from the north-central Florida community through newspaper advertising, fliers and community outreach. Participants had a mean age of 71.7 years (SD = 9.8 years) and an average of 16.26 years education (SD = 2.61, see Table 1 for detailed demographics). All participants provided written informed consent prior to enrollment. All study procedures were approved by the University of Florida Institutional Review Board prior to the start of the study. Participants had the opportunity to ask the researchers any questions about study procedures prior to the start of the study. No vulnerable populations were studied. Exclusionary criteria included pre-existing neurological or psychiatric brain disorders, MRI contraindications (such as metal or medical devices inside the body not approved to be scanned at 3T), reported diagnosis of a neurodegenerative brain disease (i.e., dementia or Alzheimer’s) or self-reported difficulty with thinking and memory.

**Table 1:** **Sample demographics**.

	*Mean*	*SD*	*Range*
Age	71.69	9.45	43–85
Education	16.26	2.61	12–20

**Sex distribution**

	*Number*	% *of sample*

Male	37	40
Female	56	60

### Study Procedures

Participants completed a neuropsychological battery (see “Measures” Section for more details) that included the NIHTB-CB and MoCA. Neuropsychological tasks were administered at an onsite clinical research facility by trained study staff. The neuropsychological battery was completed as a single visit. Neuroimaging scanning was completed at a subsequent MRI visit.

### Measures

#### NIH Toolbox

In this study, NIH Toolbox was used as a brief, comprehensive assessment to examine neurological and behavioral function, allowing for the study of functional changes across the lifespan. The cognitive domain measure was used which covered subdomains of: executive function and attention, episodic memory, language, processing speed and working memory. Executive function and attention was measured by NIH-Toolbox Flanker Inhibitory Control and Attention test and the Dimensional Change Card Sort test. Flanker measures the ability to inhibit visual attention to irrelevant task dimensions. The Dimensional Card Sort test was used to assess the set-shifting component of executive function. Working memory was tested by the List Sorting test. Episodic Memory was assessed by Picture Sequence memory test and the Auditory Verbal Learning (Rey) test. To test language, the Oral Reading Recognition test and the Picture Vocabulary test were used. Processing speed was assessed by the Pattern Comparison test and the Oral Symbol Digit test. The fluid cognition composite is composed of the following tasks: Dimensional Change Card Sort, Flanker, Picture Sequence Memory, List Sorting and Pattern Comparison. The crystalized cognition composite is composed of the Picture Vocabulary Test and the Oral Reading Recognition Test. The NIH toolbox cognitive battery has been shown to have high test-retest reliability, as well as high convergent validity with “gold standard” measures of crystalized and fluid cognition (Heaton et al., 2014).

#### MoCA

The MoCA is a 10-min, 30-point clinical assessment of multiple cognitive functions, including orientation (6 points), attention (6 points), short-term memory recall (5 points), abstract thinking (2 points), visuospatial executive function assessed by a clock-drawing task, trails task and reproducing a geometrical figure (5 points), naming task (3 points) and language function assessed by verbal fluency test (3 points). An additional one point was added for subjects with less than/equal to 12 years in education (per guidelines of MoCA administration (Nasreddine et al., 2005)). The suggested cut-off point on the MoCA is below 26 for MCI.

### Neuroimaging Acquisition

All participants were imaged in a Philips 3.0 Tesla (3T) scanner (Achieva; Philips Electronics, Amsterdam, Netherlands) at the McKnight Brain Institute (University of Florida, Gainesville, FL, USA) with a 32-channel receive-only head coil. A pillow was placed under the head to limit motion during the scan. A high-resolution 3D T1 weighted MPRAGE scan was performed. Scanning parameters consisted of: voxel size = 1 mm isotropic; 1 mm slice thickness; TE = 3.2 ms; TR = 7.0 ms; FOV = 240 × 240; Number of slices = 170; acquired in a sagittal orientation.

### Neuroimaging Processing

**Figure 1:**
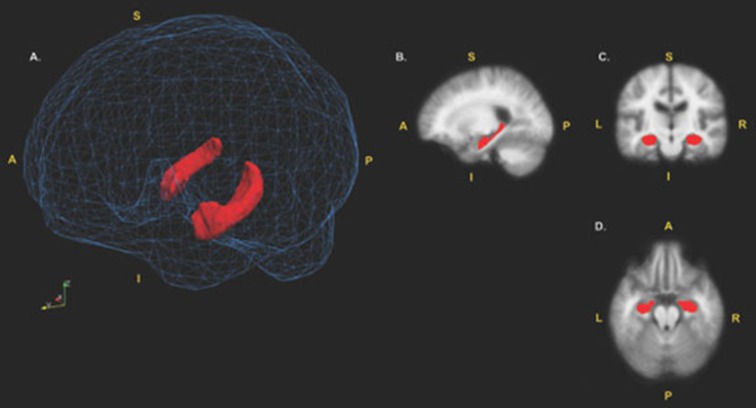
**Hippocampal region of interest (ROI). (A)** surface models of the left and right hippocampi ROIs displayed in red. The hippocampi are visualized inside a wireframe mesh provided by Madan (2015). **(B)** Sagittal view (*x* = 105), **(C)** coronal view (*z* = 110) and **(D)** axial view (*y* = 151) of the hippocampi displayed in red. Coordinates are in MNI305 space. A, Anterior; P, Posterior; I, Inferior; S, Superior.

*T*_1_-weighted MRI scans were processed with the software FreeSurfer version 5.3. To measure hippocampal volume, the automated subcortical segmentation stream in FreeSurfer was used. The software uses Bayesian inference methods relying on prior anatomical probabilities in a labeled data set, along with* a priori* known *T*_1_ intensity characteristics of subcortical regions, as well as *T*_1_ intensity information from the scan being processed, in order to label discrete regions (Fischl et al., 2002). Previous research has shown this automated procedure produces accurate and reliable results, while taking a fraction of the time of the gold standard of manual segmentation (Fischl et al., 2002; Jovicich et al., 2009). This makes automated segmentation well suited for large samples. Any errors in segmentation were fixed manually, and were re-processed through FreeSurfer, producing results that have been validated against manual segmentation (Morey et al., 2009) and histological measures (Cardinale et al., 2014). Whole hippocampal volume was computed as a sum of left and right hemisphere measures; this measure was then normalized in respect to total intracranial volume. All subsequent uses of the term “hippocampal volume” refer to the normalized value. See Figure 1 for a visual depiction of the hippocampal region of interest (ROI; mesh provided by Madan, 2015).

### Statistical Analyses

Neuroimaging data was analyzed using a ROI approach predicting hippocampal volume. MoCA and NIHTB-CB composite scores were used as predictor variables. Descriptive statistics and inter-measure correlations can be found in Tables 2, 3. Hippocampal volume was normalized using estimated total intracranial volume, to control for differences in head size. Covariates of sex and education years were included in all models. A secondary set of analyses was aimed at examining the sub-scales of MoCA and NIH toolbox fluid cognition composite to determine whether a sub-scale was driving the relationship in the total score. Due to the characteristics of MoCA, certain sub-scales did not lend themselves to further analyses. Naming, Language, Abstraction and Orientation sections were excluded due to a restriction of range in observation (i.e., a 1 point scale) and/or a lack of variability. Two subjects were excluded as outliers because they had values greater than 2.5 the standard deviation from the mean (1 hippocampal volume outlier; 1 crystallized cognition outlier).

**Table 2:** **Descriptive statistics**.

Measure	*Mean*	*SD*	*Range*
NIH Toolbox crystalized cognition	127.54	11.05	100–154
NIH Toolbox fluid cognition	96.21	9.72	80–133
MoCA	25.74	2.53	20–30

*SD, Standard Deviation*.

**Table 3:** **Correlation matrix**.

	MoCA	Crystal	Fluid
MoCA	1	0.37**	0.42**
Crystal	0.37**	1	0.28**
Fluid	0.42**	0.28**	1

Crystal, NIH Toolbox crystallized cognition; Fluid, NIH Toolbox fluid cognition. **p < 0.01.

## Results

### NIH Toolbox

#### Relationship Between NIH Toolbox Fluid Cognition and Neuroimaging Measures

**Figure 2:**
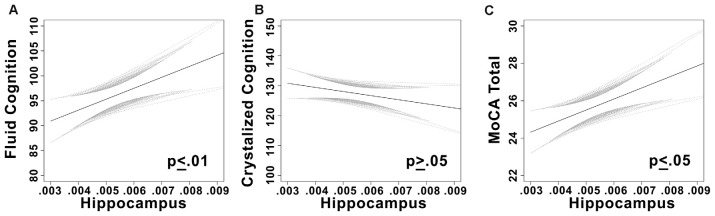
**(A)** Plots the linear relationship between fluid cognition and hippocampal volume. **(B)** Plots the linear relationship between crystalized cognition and hippocampal volume. **(C)** Plots the linear relationship between Montreal Cognitive Assessment (MoCA) total score and hippocampal volume. Confidence bands are 95% confidence intervals of the regression line.

There was a significant positive linear relationship between hippocampal volume and fluid cognition composite score (*t* = 3.3, *p* = 0.001, partial *η*^2^ = 0.11; see Figure 2) while controlling for sex and years of education (full model (*F*_(3,89)_ = 3.81, *p* = 0.013, *r*^2^ = 0.11).

#### Relationship Between NIH Toolbox Crystallized Cognition and Neuroimaging Measures

As expected, no relationship was observed between hippocampal volume and crystallized cognition while controlling for covariates (*t* = −0.21, *p* = 0.84). Univariate models were nonsignificant as well; neither composite nor individual sub-scales of crystallized cognition were significantly related to hippocampal volume (*p’s* > 0.05).

#### Relationship Between NIH Toolbox Sub-Scales and Neuroimaging Measures

**Figure 3:**
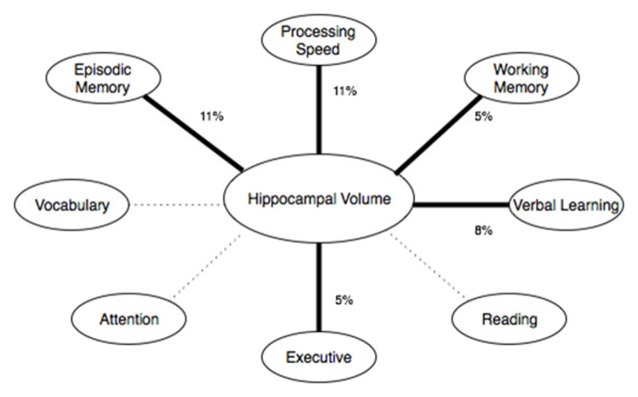
**Shows the cognitive domains of the NIH toolbox and their relationship with hippocampal volume.** Thick, solid lines represent significant (*p* < 0.05) effects. Dashed lines represent non-significant effects. The percentage represents the effect size (percent variance explained by the predictor variable).

Five linear models were analyzed using each sub-scale of the NIH toolbox fluid cognition composite while controlling for sex and years of education. Dimensional Change Card Sorting (*p* = 0.027, partial *η*^2^ = 0.05, observed power = 0.60), Picture sequence memory (*p* = 0.001, partial *η*^2^ = 0.11, observed power = 0.90), List Sorting (*p* = 0.04, partial *η*^2^ = 0.05, observed power = 0.54) and Pattern comparison (*p* = 0.002, partial *η*^2^ = 0.11, observed power = 0.89) were predicted by hippocampal volume. The attention domain flanker task was not significantly (*p* > 0.25) related to hippocampal volume. The strongest predictor of hippocampal volume was the pattern comparison task, which is in the processing speed domain. The episodic memory (picture sequence memory task) domain and the working memory domain (list sorting task) were both significantly related to hippocampal volume. Two supplemental tasks, the symbol digit search and Rey verbal learning were also analyzed (these tasks do not factor into the fluid cognition composite score, but were including in our NIH-Toolbox cognitive module). Rey verbal learning (*p* = 0.008, partial *η*^2^ = 0.08, observed power = 0.77) and symbol digit search (*p* = 0.073, partial *η*^2^ = 0.04, observed power = 0.44) scores showed a positive relationship with hippocampal volume. See Figure 3 for a summary.

### MoCA

#### Relationship Between MoCA and Neuroimaging Measures

There was a significant positive linear relationship between hippocampal volume and total MoCA score while controlling for sex and years of education (*t* = 2.36, *p* = 0.02, partial *η*^2^ = 0.06).

#### Relationship Between MoCA Subscales and Neuroimaging Measures

Three subscales of the MoCA were analyzed individually: delayed recall, attention and visual-spatial/executive. As hypothesized, delayed recall was associated with hippocampal volume (*t* = 1.96, *p* = 0.052). No associations were found between attention (*t* = 0.75, *p* = 0.454) or visual-spatial/executive (*t* = 0 0.88, *p* = 0.382).

## Discussion

Hippocampal volume predicted cognitive performance on both the MoCA and NIH toolbox fluid cognition composite score in a community sample of 93 older adults without clinical history of MCI, neurodegenerative disease, neurological injury or self-reported memory problems. This finding supports the study hypothesis that smaller hippocampal volume is associated with poorer cognitive performance in older adults, particularly with respect to memory-related functions. A relationship in hippocampal volume was found only for fluid abilities, and not crystallized abilities such as vocabulary or reading. This disassociation has been described in patient studies such as HM, where bilateral hippocampal resection caused profound memory disturbances while general knowledge remained intact (Scoville and Milner, 1957).

Prior literature demonstrates a strong relationship between the hippocampus and learning, memory and other fluid cognitive functions in both animals and humans (Raz et al., 1998; Petersen et al., 2000), with smaller volumes associated with poorer performance (Persson et al., 2006). However, these results have not been universal in older adults (Van Petten, 2004). Our data not only demonstrate a strong relationship between hippocampal volume and episodic memory, but also relationships with executive function, working memory and speed of processing.

### Cognitive Subdomains and Hippocampal Volume

Within the MoCA and NIHTB, subtests that targeted the memory domain were significantly related to hippocampal volume. This replicates previous research showing a positive relationship between hippocampal atrophy and memory measures in non-demented subjects (Golomb et al., 1996; Persson et al., 2006). However, as mentioned, not all studies have replicated this finding. A meta-analysis by Van Petten (Van Petten, 2004) suggested that overall evidence in the literature for a positive relationship between hippocampal size and episodic memory in older adults was “surprisingly weak.” In addition, a prior study investigating the relationship between hippocampal volume and MoCA failed to find such a relationship (Paul et al., 2011). While the current study and Paul et al. (2011) were similar in statistical power, imaging methods, and study inclusion/exclusion criteria, our sample was approximately 10 years older. As the relationship between hippocampal volume and memory is non-linear with age, this difference in our cohort’s average age may account for the difference in our findings (Chen et al., 2016).

Regardless, this study is the first to report a significant positive relationship between hippocampal volume, MoCA and NIHTB-CB memory measures. However, caution in the interpretation of these findings is warranted. “Bigger is better” is certainly an oversimplification; smaller hippocampi have been associated with better memory in children and adolescents (Sowell et al., 2001). Furthermore, in pathological conditions, such as Fragile X syndrome, enlarged hippocampi are associated with poorer memory performance (Molnár and Kéri, 2014). The biological change associated with increased or decreased brain volume could be the result of multiple processes, which we are unable to elucidate with T1 structural MRI techniques. For example, increased volume could be the result of increased neuronal cell bodies, increases in glia or astrocytes, neuroinflammation or insufficient neuronal pruning. Nonetheless, our results demonstrate that smaller hippocampal volume is associated with decreased performance on two well validated and commonly administered measures of cognitive function in older adults, with particular sensitivity to memory function across both tasks.

Hippocampal volume was associated with performance in other cognitive domains besides memory on the NIHTB Cognitive Battery, specifically speed of processing, working memory and executive function (see Figure 3). In fact, the association between hippocampal volume and processing speed on the NIHTB was slightly stronger than for episodic memory. As delayed recall is not assessed by the NIH-Toolbox, it is possible that the relationship with delayed recall observed on the MoCA was not detectable from the NIH-Toolbox Cognitive Battery. Regardless, our results highlight the multifaceted role of the hippocampus in cognitive aging. The hippocampus contributes to other cognitive functions besides memory, and optimal learning and memory depends on other cognitive functions, such as working memory, processing speed and executive functioning, in addition to encoding and storage. A relationship between speed of processing and hippocampal volume has been shown in some (Tisserand et al., 2000), but not all past studies (Colom et al., 2013). An association between hippocampal volume and executive functioning was only evident on the NIH toolbox (dimension change card sorting), not for the MoCA executive-visual spatial sub-scale. Dimensional Change Card Sorting has greater cognitive demand and requires higher-order executive processes compared to the MoCA executive tasks. For example, the Dimensional Change Card Sorting task would require effort from multiple CHC factors, such as fluid reasoning, short-term memory, visual processing and reaction speed. Even though the executive tasks in NIH Toolbox and MoCA are classified as part of the same domain, performance on these tasks was not correlated (*r* = 0.07, *p* > 0.05). This supports the conclusion that these tests measure different elements of executive functioning. While the relationship between fluid cognition and hippocampal volume may seem surprising due to the traditional association of fluid abilities (particularly executive function and processing speed) and the pre-frontal cortex, previous studies have implicated hippocampal volume as a predictor of fluid ability in older adults (while no such association was found in younger adults; Reuben et al., 2011). A potential mechanism of the hippocampal association with fluid ability in older adults may relate to compensatory processes in the hippocampus as a result of the pre-frontal atrophy observed with age. Further research, particularly longitudinal studies, are needed to clarify whether the relationship between hippocampal volume and fluid ability changes throughout the lifespan, and which potential mechanisms may account for such change.

## Conclusion

Prior research has produced controversy over the role of the hippocampus in cognitive aging, casting doubt on its role in episodic memory, as well as other domains (e.g., speed of processing, Van Petten, 2004; Colom et al., 2013). Our findings demonstrate that the hippocampus is a critical structure in cognitive aging, playing a role not only in episodic memory, but also processing speed, working memory and executive function. Whether effects of the hippocampus on domains outside of episodic memory are direct or meditational in nature remains to be seen. Our findings also demonstrate that performance on a commonly used bedside dementia screening (MoCA) and a comprehensive cognitive battery (NIH Toolbox) are significantly related to hippocampal volume. Whereas, a prior study failed to find a relationship between MoCA and hippocampal volume in an older adult population (Paul et al., 2011), we found that our cohort, approximately 10 years senior in average age, evidenced a significant relationship. These data suggest a foundation for longitudinal research investigating hippocampal volume in older adults as a possible predictor of future decline or MCI conversion. Such data would help to elucidate issues of acute vs. progressive atrophy in the study, and further clarify potential implications for pathologies like MCI and AD. More importantly, our data provide strong evidence in support of the multifaceted role of the hippocampus in cognitive aging.

## Author Contributions

AO, RAC, ECP, NRN and AJW contributed text to the manuscript. AO and AJW performed data analysis. All authors provided edits and approved the final version of the manuscript.

## Funding

AJW and AO are partially supported by the McKnight Brain Research Foundation and the University of Florida Cognitive Aging and Memory Clinical Translational Research Program. AJW is partially supported by the NIH/NCATS CTSA grant UL1 TR000064 and KL2 TR000065, NIA K01AG050707-A1 and R01AG054077. The funding sources had no involvement in the study design, collection, analysis and interpretation of the data, in the writing of the manuscript and in the decision to submit the manuscript for publication. Neuroimaging was performed at the Advanced Magnetic Resonance Imaging and Spectroscopy (AMRIS) facility in the McKnight Brain Institute of the University of Florida, which is supported by National Science Foundation Cooperative Agreement no. DMR-1157490 and the State of Florida.

## Conflict of Interest Statement

The authors declare that the research was conducted in the absence of any commercial or financial relationships that could be construed as a potential conflict of interest. The handling Editor declared a shared affiliation, though no other collaboration, with the authors and states that the process nevertheless met the standards of a fair and objective review.
